# Characterization and identification of PARM-1 as a new potential oncogene

**DOI:** 10.1186/1476-4598-12-84

**Published:** 2013-07-31

**Authors:** Cyndia Charfi, Louis-Charles Levros, Elsy Edouard, Eric Rassart

**Affiliations:** 1 Laboratoire de Biologie Moléculaire, Département des Sciences Biologiques, Centre BioMed, Université du Québec à Montréal, Case Postale 8888, Succursale Centre-ville, Montréal, QC H3C-3P8, Canada

**Keywords:** T leukemia, Gene profiling, Graffi MuLV, PARM-1, Oncogene

## Abstract

**Background:**

The Graffi murine retrovirus is a powerful tool to find leukemia associated oncogenes. Using DNA microarrays, we recently identified several genes specifically deregulated in T- and B-leukemias induced by this virus.

**Results:**

In the present study, probsets associated with T-CD8^+^ leukemias were analyzed and we validated the expression profile of the *Parm-1* gene. PARM-1 is a member of the mucin family. We showed that human PARM-1 is an intact secreted protein accumulating predominantly, such as murine PARM-1, at the Golgi and in the early and late endosomes. PARM-1 colocalization with α-tubulin suggests that its trafficking within the cell involves the microtubule cytoskeleton. Also, the protein co-localizes with caveolin-1 which probably mediates its internalization. Transient transfection of both mouse and human Parm-1 cDNAs conferred anchorage- and serum-independent growth and enhanced cell proliferation. Moreover, deletion mutants of human PARM-1 without either extracellular or cytoplasmic portions seem to retain the ability to induce anchorage-independent growth of NIH/3T3 cells. In addition, PARM-1 increases ERK1/2, but more importantly AKT and STAT3 phosphorylation.

**Conclusions:**

Our results strongly suggest the oncogenic potential of PARM-1.

## Background

The Graffi murine leukemia virus (MuLV) induces a wide spectrum of leukemias in several strains of mice, including lymphoid and non-lymphoid types [1,2] making of this virus a good model to gain new insights on lymphoid leukemia development and progression and to identify new oncogenes. Retroviruses have been used as molecular tools to identify oncogenes or tumor suppressors directly targeted through the retroviral integration. However the microarray technology is attractive because it allows identifying, in addition to the retrovirus targeted genes, those involved in the cascade of events that leads to cell transformation, tumor progression, cancer and metastasis. We therefore used this approach to compare the transcriptome of a full panel of leukemias induced by the Graffi MuLV [2] and we focused our analyses on the lymphoid types [3]. We identified genes that were deregulated in one type of leukemia when compared to the corresponding control, therefore representing potential markers and oncogenes or tumor suppressor candidates that are specific for B, T or common to both types of leukemia. As expected, many of these genes were known to be specific to a lineage and to leukemia types (for details, see: [4]). Furthermore, we validated changes in the expression levels of 10 genes selected according to their specificity for lymphoid leukemias. These results clearly validated our approach and identified genes that now deserve more attention. Indeed, we previously reported that the *Fmn2* gene harbors oncogenic potential. It was found specifically over-expressed in murine B-leukemias as well as in human pre-B-ALL especially in children bearing a t(12;21) translocation (TEL/AML1 rearrangement) [3].

In this study, we focused on genes that are associated with T-CD8^+^ leukemias. We identified *Parm-1* (prostate androgen-regulated mucin-like protein 1), a gene specifically up-regulated in T-CD8^+^ leukemias induced by Graffi virus. PARM-1 is a member of the mucin family. Very little is known about the physiological and biological function of this gene and its precise role in cellular transformation has not been fully explored.

We characterized the function of PARM-1 and we investigated the oncogenic potential of mouse and human proteins. PARM-1 is a weakly secreted protein which contains a transmembrane domain (TM) and a cytoplasmic tail (CT) in addition to the extracellular (EC) domains. Both human (hPARM-1) and mouse (mPARM-1) proteins are predominantly located at the Golgi and in the early and late endosomes but transiently located at the plasma membrane. PARM-1 trafficking within the cells seems associated with the microtubule cytoskeleton. Also, PARM-1 induced both anchorage and serum-independent growth, enhanced cell proliferation and activated ERK1/2, AKT and STAT3.

Together, these results provide strong evidences for the oncogenic potential of PARM-1 and emphasize their important role in leukemogenesis.

## Results

### Microarray data analyses and validation of mParm-1 association with T-CD8^+^ leukemias

In our previous study, to gain insight into the cancerous signatures of lymphoid leukemias, the gene expression profile of three T-leukemias and of three B-leukemias induced by the Graffi MuLV was analyzed using microarrays technology and compared to those of non-leukemic B- and T-cells, respectively [3]. We identified a set of genes that are specific markers for Graffi MuLV-induced B and T leukemias. In this study, we focused on genes that were only associated with T-CD8^+^ leukemias. Accordingly, 42 probsets (32 genes) were over-expressed and 8 probsets (7 genes) were down-regulated. Some were already associated with T-CD8^+^ leukemias (*Il2ra *[5]; and *Pdgfrb *[6]) and others were associated with other types of T-leukemias or cancer (*Irf4 *[7], *Hrb *[8], *Depdc6 *[9], *Als2cl *[10], *Tle4 *[11] and *Cdc42ep3 *[12]) (Table 1), thus validating our approach. Interestingly, many other genes were neither associated with leukemias nor with other types of cancer, or had no assigned function representing therefore good candidates as specific markers, oncogenes or tumor suppressors for T-CD8^+^ leukemias. The complete list of these probsets is presented in Table 1.

**Table 1:** **Probsets associated with T CD8**^**+**^**leukemias**

**ProbesetIDs**	**T1-CT**	**T2-CT**	**T3-CT**	**B1-CT**	**B2-CT**	**B3-CT**	**Gene Title**	**Gene Symbol**
	*	*	*	*	*	*		
**T CD8+ overexpressed genes**								
1420692_at	5,83	5,52	−0,02	−0,21	0,12	0,52	interleukin 2 receptor, alpha chain	*Il2ra*
1456645_at	5,75	5,21	0,85	−0,23	−0,14	−0,14	WD repeat domain 25	*Wdr25*
1435436_at	5,70	5,77	0,77	−0,02	−0,05	0,28	Transcribed locus	*—*
1427802_a_at	5,63	7,13	−0,14	0,53	−0,22	−0,33	*—*	*—*
1436970_a_at	5,35	7,13	0,87	0,22	−0,73	0,01	platelet derived growth factor receptor, beta polypeptide	*Pdgfrb*
1428420_a_at	4,88	4,98	−0,12	−0,19	−0,42	−0,19	RIKEN cDNA 1200009I06 gene	*Exoc3l4*
1419302_at	4,39	4,77	0,10	0,65	0,51	0,17	hairy/enhancer-of-split related with YRPW motif-like	*Heyl*
1440808_x_at	4,20	3,61	0,02	−0,62	−0,45	−0,58	CD163 molecule-like 1	*Cd163l1*
1420691_at	4,19	4,39	−0,17	−0,10	0,12	0,45	interleukin 2 receptor, alpha chain	*Il2ra*
1424214_at	4,07	4,78	0,28	−0,62	−0,93	−0,83	RIKEN cDNA 9130213B05 gene (prostate androgen-regulated mucin-like protein 1)	*9130213B05Rik (Parm-1*
1459461_at	4,04	5,39	0,24	0,54	0,67	0,44	—	—
1428891_at	4,01	4,27	0,29	−0,22	−0,18	−0,26	RIKEN cDNA 9130213B05 gene (prostate androgen-regulated mucin-like protein 1)	*9130213B05Rik (Parm-1*
1460294_at	3,84	4,65	0,16	−0,20	−0,20	0,15	ATPase, aminophospholipid transporter-like, class I, type 8A, member 2	*Atp8a2*
1440156_s_at	3,84	3,80	0,51	−0,13	−0,28	−0,35	TOX high mobility group box family member 2	*Tox2*
1435494_s_at	3,76	7,29	0,37	0,07	−0,12	0,00	desmoplakin	*Dsp*
1455527_at	3,59	3,34	0,29	−0,25	−0,30	−0,16	CD163 molecule-like 1	*Cd163l1*
1444696_at	3,34	3,50	−0,04	0,22	−0,15	0,18	Transcribed locus, weakly similar to NP_001311.3 casein kinase 2, beta polypeptide [Homo sapiens]	
1421173_at	3,33	2,23	0,17	−0,63	−0,10	−0,60	interferon regulatory factor 4	*Irf4*
1440627_at	3,31	5,10	0,10	−0,19	−0,49	−0,47	ATPase, aminophospholipid transporter-like, class I, type 8A, member 2	*Atp8a2*
1446224_at	3,22	3,00	0,97	−0,13	−0,26	−0,26	HECT domain containing 2	*Hectd2*
1438886_at	3,12	3,09	0,29	−0,03	−0,22	0,13	hairy/enhancer-of-split related with YRPW motif-like	*Heyl*
1459219_at	3,09	5,30	−0,36	0,32	0,51	0,14	RIKEN cDNA B930012P20 gene	*Rapgef2*
1426922_s_at	3,06	3,55	0,45	0,25	0,63	0,12	HIV-1 Rev binding protein	*Hrb*
1433906_at	3,01	5,03	0,00	−0,01	−0,05	0,10	Clavesin 1	*Clvs1*
1450897_at	2,81	3,83	−0,62	−0,44	−0,62	−0,46	expressed sequence AU014947	*AU014947*
1451348_at	2,74	4,18,	−0,55	0,77	0,58	0,38	DEP domain containing 6	*Depdc6*
1452237_at	2,71	3,29	0,38	−0,11	0,15	−0,02	HIV-1 Rev binding protein	*Hrb*
1440870_at	2,56	3,69	−0,33	0,01	0,00	0,10	PR domain containing 16	*Prdm16*
1426923_at	2,46	2,90	0,14	−0,07	0,23	−0,01	HIV-1 Rev binding protein	*Hrb*
1421508_at	2,45	2,02	−0,78	−0,44	−0,63	−0,28	odd Oz/ten-m homolog 1 (Drosophila)	*Odz1*
1424814_a_at	2,45	2,31	0,59	−0,41	−0,60	−0,95	Bcl2-like 14 (apoptosis facilitator)	*Bcl2l14*
1452238_at	2,44	3,31	0,09	0,24	0,28	−0,04	HIV-1 Rev binding protein	*Hrb*
1423194_at	2,43	2,44	−0,27	−0,89	−0,57	−0,68	—	—
1441548_at	2,39	2,76	−0,23	0,04	−0,21	−0,25	FERM domain containing 4B	*Frmd4b*
1456956_at	2,36	3,95	0,56	−0,31	−0,59	−0,94	IKAROS family zinc finger 2	*Ikzf*
1434186_at	2,29	2,86	−0,40	0,00	−0,08	0,18	G protein-coupled receptor 23	*Gpr23*
1428622_at	2,23	3,86	0,08	0,66	0,66	0,75	DEP domain containing 6	*Depdc6*
1455449_at	2,19	4,11	−0,22	0,17	0,33	0,18	RIKEN cDNA 2010107G12 gene	*Gm468*
1423666_s_at	2,13	2,18	0,90	0,82	0,88	0,35	ribosomal protein L5	*Rpl5*
1430029_a_at	2,04	2,22	−0,44	0,31	0,24	0,24	sarcoma amplified sequence	*Sas*
1437542_at	2,02	2,39	0,87	0,38	0,21	−0,15	IKAROS family zinc finger 2	*Ikzf2*
1427132_at	2,00	2,18	0,42	−0,62	−0,36	−0,81	myotubularin related protein 13	*Mtmr13*
**T CD8+ underexpressed genes**								
1433769_at	−2,00	−4,28	0,03	−0,21	−0,26	−0,31	ALS2 C-terminal like	*Als2c*
1424338_at	−2,20	−2,20	0,62	−0,16	−0,98	0,20	solute carrier family 6 (neurotransmitter transporter, GABA), member 13	*Slc6a13*
1420918_at	−3,43	−2,50	−0,26	−0,72	0,07	−0,01	serum/glucocorticoid regulated kinase 3	*Sgk3*
1450455_s_at	−3,49	−3,04	−0,73	−0,29	−0,22	0,09	aldo-keto reductase family 1, member C12 /// aldo-keto reductase family 1, member C13	*Akr1c12 /// Akr1c13*
1420919_at	−3,54	−2,36	−0,18	−0,01	0,44	0,49	serum/glucocorticoid regulated kinase 3	*Sgk3*
1450853_at	−3,59	−2,77	0,36	0,28	0,04	0,74	transducin-like enhancer of split 4, homolog of Drosophila E(spl)	*Tle4*
1452657_at	4,09	2,04	0,29	0,17	−0,67	−0,87	adaptor-related protein complex 1, sigma 2 subunit	*Ap1s2*
1450700_at	−4,12	−2,08	−0,86	0,64	0,28	0,70	CDC42 effector protein (Rho GTPase binding) 3	*Cdc42ep3*

Results are presented in log base 2. A positive deviation of 4 and above and a negative deviation of 4 and below means a fold-change of 4. Values comprised between 0,585 and −0,585.

*ratio T-CT and B-CB: mean of the deviation of T1, T2 and T3/T control value and mean of the deviation of B1, B2 and B3/B control value.

**Figure 1:**
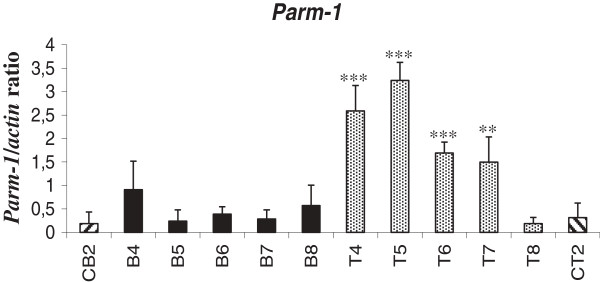
**Analysis of m*Parm-1 *gene expression in sorted lymphoid leukemia samples.** Semi-quantitative RT-PCR analysis of *Parm-1* in 5 B and 5 T leukemias : (B4, Cd45^+^Cd19^+^Sca1^+^; B5, Cd45R^+^Cd19^+^Sca1^+^; B6, Cd45R^+^Cd19^+^Sca1^+^; B7, Cd45R^+^Cd19^+^Sca1^+^; B8, Cd45R^+^Cd19^+^Sca1^+^) (T4, Cd4^+^Cd8^+^; T5, Cd4^+^Cd8^+^; T6, Cd4^+^Cd8^+^; T7, Cd4^+^Cd8^+^; T8, Cd4^+^Cd8^-^). RT-PCRs were performed in triplicate using the following actin (forward primer: 5’- tgacggggtcacccacactgtgcccatcta-3’, reverse primer: 5’-ctagaagcatttgcggtggacgatggaggg-3’) and murine Parm-1 (forward primer: 5’- gttagctgttttggggacca-3’, reverse primer: 5’-cgtgcaaattagcatctgga-3’) specific primers. PCR products were analyzed on agarose gel and band density was quantified with Quantity One Image Software. The actin gene was used as internal control and expression levels in each leukemia are presented as a gene/actin density ratio. Data are representative of 3 independent experiments. Statistical analysis was performed using one-way analysis of variance, and P lower to 0.05 was considered to be significant (*P ≤ 0.05, **P ≤ 0.01, ***P ≤ 0.001) compared with their respective control (CB2, B cells from normal spleen and CT2, T cells from normal thymus).

We focused on the *mParm-1* (9130213B05Rik) gene. The expression level of *mParm-1* was measured by semi-quantitative RT-PCR in several Graffi MuLV-induced tumors. Significant over-expression was only observed in T-CD8^+^ tumors when compared to control T-cells. This result confirms the specificity of the *mParm-1* gene up-regulation to T-CD8^+^ leukemias (Figure 1).

### PARM-1 sequence analysis

**Figure 2:**
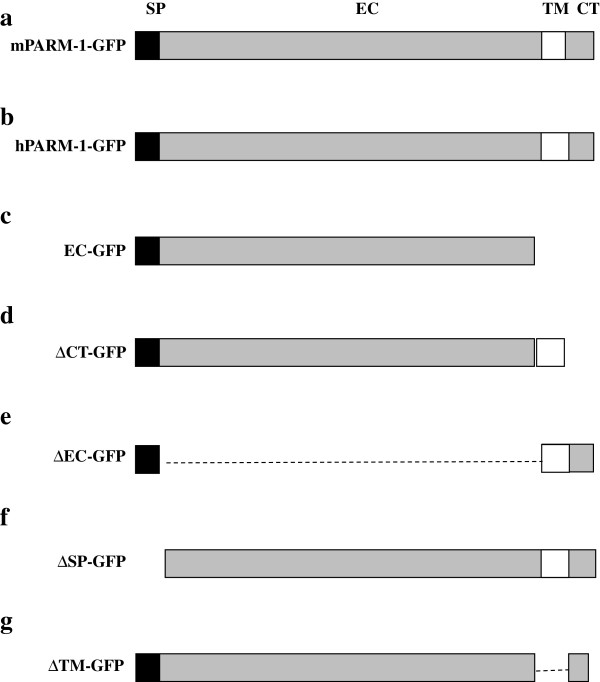
**Schematic representation of full-length and deletion mutant constructs of mPARM-1 and hPARM-1. (a and b)** Full-length constructs of the mouse (296 a.a) and human PARM-1 (310 a.a). **(c to g)** representation of the human deletion mutants. SP: signal peptide: (1–20 a.a), EC: extracellular domain (mPARM-1: 20–246 a.a; hPARM-1: 20–260 a.a), TM: transmembrane domain (mPARM-1: 246–266 a.a; hPARM-1: 260–280 a.a), and CT: cytoplasmic tail (mPARM-1: 266–296 a.a; hPARM-1: 280–310 a.a). EGFP was fused in the C-terminal end of all constructs.

PARM-1 is a member of the mucin family known to be expressed at the surface of many epithelial cells [13] to promote cell survival by protecting the cell surface and to be implicated in cancer development [14]. Protein sequence analysis of mPARM-1 showed that, as the hPARM-1 and in addition to its single transmembrane domain, mPARM-1 possess an N-terminal signal peptide (Figure 2a and 2b) [15]. mPARM-1 sequence contains 3 N-glycosylated motifs and 65 mucin-type O-glycosylated sites [16], suggesting that, as its human counterpart, mPARM-1 should be highly glycosylated. Moreover, we found that 41% of the amino acid composition of mPARM-1 is represented by serine, proline and threonine residues similar to the human protein [17]. Interestingly, amino acid sequence alignment of PARM-1 homologs showed that the C-terminus is highly conserved (Additional file 1: Figure S1) suggesting an important role through evolution.

### PARM-1 protein characterization

**Figure 3:**
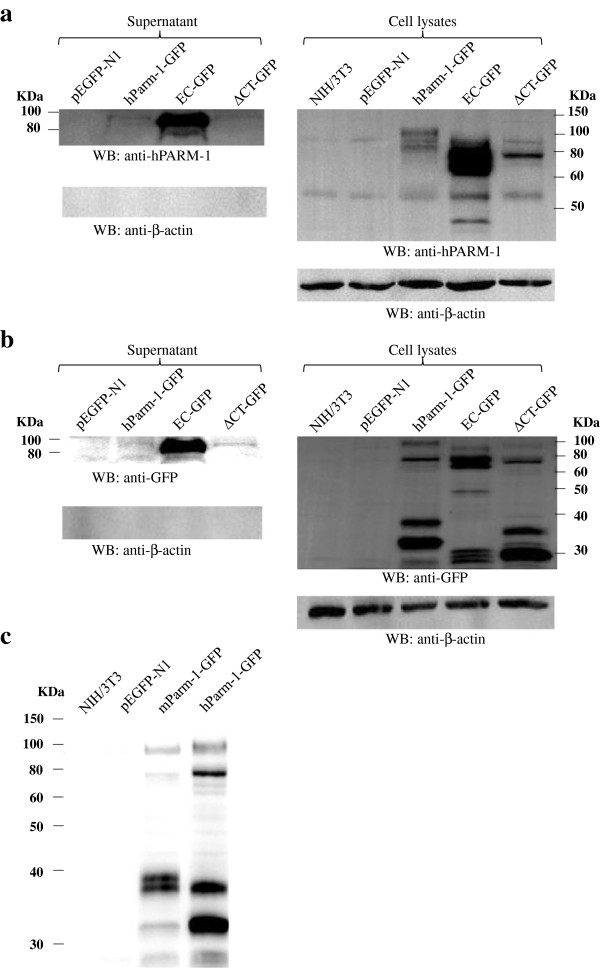
**PARM-1 protein profile and secretion by NIH/3T3 cells.** Immunoblotting of lysates (30 μg) from NIH/3T3 cells transiently transfected with expression vector of hParm-1 (full-length or mutants) or of mParm-1 using **(a)** an anti-hPARM-1 (Sigma; 1:1000) antibody or **(b)** an anti-GFP (Santa Cruz Biotechnology; 1:1000) antibody. Culture supernatants from these cells were collected, centrifuged, concentrated and subjected to SDS-PAGE (12 %) and hPARM-1 protein was detected by western blot **(a)** using anti-hPARM-1 and **(b)** anti-GFP antibodies recognizing respectively the N-terminus and C-terminus of hPARM-1 fusion protein. The region below 80-kDa from the supernatant in panels **a** and **b** contains no detectable bands. The anti-β-actin (Sigma; 1:1000) was used to detect possible media contaminations by proteins from lysed cell. **(c)** mPARM-1 expression was also tested in cell lysates.

The EC domain of most transmembrane mucins is released from the cell surface and we verified if this was the case for PARM-1. Culture supernatant of NIH/3T3 cells transfected with hParm-1-GFP was collected and the presence of hPARM-1 visualized by western blot using either anti-hPARM-1 (specific for the EC portion) or anti-GFP antibodies (specific for the GFP tag in C-terminal). Lysates from NIH/3T3 expressing hPARM-1-GFP were also analyzed. Using the anti-hPARM-1 antibody, hPARM-1-GFP was detected in the supernatant as a very faint band slightly lower than 100-kDa. We then used two deletion mutant constructs, one deleted for the TM and CT domains (EC-GFP, Figure 2c) and the other missing only the CT portion (∆CT-GFP, Figure 2d) of hPARM-1. Our results showed that ∆CT-GFP mutant protein was also secreted in approximately the same proportion and size as the full-length construct. However, the EC-GFP mutant was found to be secreted as two bands: one intense band of about 90-kDa and a weaker band of about 70-kDa (Figure 3a; See Additional file 2: Figure S2 to compare the molecular weight of all the obtained bands). The abundance of EC-GFP in both the cell lysate and the supernatant probably reflects protein stability (Figure 3a and Additional file 2: Figure S2). Surprisingly, anti-GFP antibodies detected the secreted protein for the three constructs at the same molecular weight as for the anti-hPARM-1 antibodies (Figure 3b and Additional file 2: Figure S2) suggesting that the protein could be entirely secreted since the GFP tag is located at the C-terminal end. We could not detect actin in these supernatants excluding contamination from lysed cells. These results suggest that PARM-1 is a secreted intact protein.

Using the anti-GFP antibody, we noted a more complex expression pattern of hPARM-1-GFP in the lysates from NIH/3T3 transfected cells than that obtained with the anti-hPARM-1 antibody. Indeed, for the hParm-1-GFP construct, in addition to the 2 bands of about 80-kDa and 120-kDa (probably a highly glycosylated and homodimeric forms, respectively) detected by the anti-hPARM-1 antibody, two other intense bands with a lower size (between 30 and 40-kDa) were detected by the anti-GFP antibody. These bands may result from a cleavage liberating the C terminus of hPARM-1 (Figure 3a and 3b and Additional file 2: Figure S2). Similar result was obtained for the cell lysates of NIH/3T3 transfected with mParm-1-GFP. Using anti-GFP antibodies, five bands were obtained: one over 100-kDa, one of about 80-kDa, and three between 30 and 40-kDa (Figure 3c and Additional file 2: Figure S2). Unfortunately, the anti-hPARM-1 was not able to recognize the murine protein.

### PARM-1 colocalizes with the Golgi apparatus and with early and late endosomes

**Figure 4:**
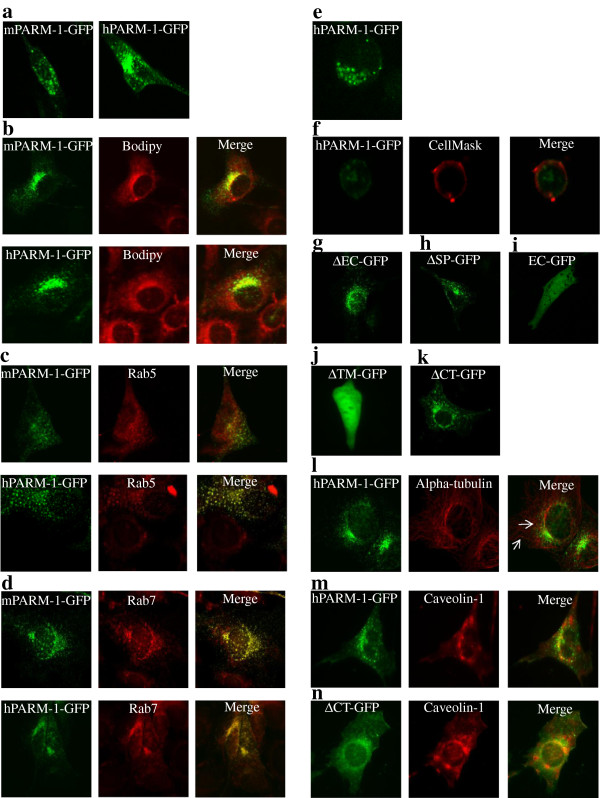
**Subcellular localization of mPARM-1 and hPARM-1 (full-length and mutant proteins). (a)** NIH/3T3 cells, transiently transfected with GFP-tagged mParm-1 or hParm-1 constructs were visualized using confocal microscopy. **(b)** For the Golgi co-localization, transfected NIH/3T3 cells were fixed and stained with Bodipy ceramide marker. For late and early endosomes co-localization, fixed cells were labeled with **(c)** anti-Rab5 (early endosomes (1:100, Cell signaling)) and **(d)** anti-Rab7 (late endosomes (1:100, Cell signaling)) antibodies, respectively. Jurkat T cells, transiently transfected with hParm-1-GFP proteins were visualized **(e)** without fixation or **(f)** following fixation and staining with CellMask plasma membrane labeling. NIH/3T3 cells were transiently transfected with **(g)** ∆EC-GFP, **(h)** ∆SP-GFP, **(i)** EC-GFP, **(j)** ∆TM-GFP and **(k)** ∆CT-GFP constructs of hPARM-1 and visualized using confocal microscopy. **(l)** hPARM-1-GFP co-localizes with microtubules. NIH/3T3 cells transfected with hParm-1-GFP construct were fixed and stained with anti-α-tubulin (1/2000, Sigma) antibody. Arrows indicate co-localized hPARM-1-GFP vesicles and microtubules. NIH/3T3 cells transiently expressing **(m)** hPARM-1-GFP or **(n)** ∆CT-GFP were fixed, immunostained for caveolin-1 (1:100, Novus Biologicals), and examined by confocal fluorescence microscopy. For hPARM-1-GFP-caveolin-1 co-localization, cells that clearly demonstrated cell membrane PARM-1 localization were chosen. All co-localizations were observed following merging images of GFP-tagged proteins with those of Golgi, endosomes, plasma membrane, α-tubulin or caveolin-1 labeling. Cells were imaged with a laser-scanning confocal microscope (Bio-Rad MRC-1024 ES) mounted on a Nikon TE-300 using a Plan Apochromat 60x (NA 1.40) oil objective (Nikon), digitally acquired using Laser Sharp software Version 3.2 (Bio-Rad). For live cell imaging, signals were collected at a rate of 2 seconds. Images were analyzed using NIH ImageJ Version 1.42l software. Data are representative of 3 independent experiments.

We were interested to confirm that hPARM-1 protein is localized to the Golgi, at the early endocytic pathway and at the plasma membrane [17] and investigated the localization of the murine protein in NIH/3T3 cells. Both mPARM-1-GFP or hPARM-1-GFP proteins were localized at the Golgi and have punctate and typical endosomal localization (Figure 4a). Similar results were obtained using a Myc-tagged protein and upon transfection with much less plasmid (data not shown), indicating that neither the GFP tag, nor the over-expression of PARM-1 disturbed its localization. The Golgi colocalization was confirmed following cell staining with the bodipy Golgi marker (Figure 4b). To quantify this colocalization, the Pearson’s correlation coefficient (Rr) was calculated using the ImageJ software. The values are ranged from 1 (perfect correlation) to −1 (perfect exclusion), zero corresponding to random localization. The Rr values are 0.68 for hPARM-1-GFP and 0.74 for mPARM-1-GFP confirming the colocalization of both human and murine PARM-1 with the golgi marker. The endosomal colocalization was also confirmed following immunolabelling of cells with anti-Rab5 (hPARM-1-GFP (Rr: 0.83); mPARM-1-GFP (Rr: 0.54)) and anti-Rab7 (hPARM-1-GFP (Rr: 0.86); mPARM-1-GFP (Rr: 0.88)) antibodies (Figure 4c and 4d). Surprisingly, localization at the plasma membrane was very weak for both proteins in NIH/3T3 (Figure 4a-d) and Jurkat T-cells (Rr: 0.2) transiently transfected with hParm-1-GFP (Figure 4e) and following cell membrane marker staining (Figure 4f) demonstrating that mPARM-1 has the same localization as its human homolog.

NIH/3T3 cells were transfected with different hParm-1-GFP deletion mutants. ∆EC-GFP and ∆SP-GFP have the same localization as the hPARM-1-GFP (Figure 4g and 4h). EC-GFP and ∆TM-GFP showed a diffuse localization through all cellular compartments (Figure 4i and 4j). ∆CT-GFP showed the same localization as the full-length hPARM-1-GFP. However, this mutant is clearly localized at the plasma membrane as well as in the intracellular compartment (Figure 4k). These results suggest that the TM probably determines Golgi-endocytic pathway localization and that the CT inhibits plasma membrane localization of PARM-1.

### PARM-1 recycling

To monitor trafficking of PARM-1, NIH/3T3 cells were transfected with hPARM-1-GFP construct and subjected to live cell time-lapse microscopy. Cells incubated at 37°C showed highly motile hPARM-1-GFP vesicles, traveling very quickly inside the cell and moving from the cytoplasm to the cell surface and instantly recycled inside the cell (Additional file 3: Movie S1 (left panel)). Some particles shuttled over short distances between plasma membrane and a close compartment that may represent early endosomes suggesting a fast-recycling pathway. Some other vesicles recycled from plasma membrane and traveled over longer distances suggesting a slow-recycling pathway [18] (Additional file 3: Movie S1 (left panel)). Since low temperature are known to inhibit all active processes including endocytosis [19], transfected NIH/3T3 cells were incubated at 4°C. We showed that the motility of hPARM-1-GFP vesicles was inhibited when compared to that in cells at 37°C indicating that recycling of hPARM is energy dependent (Additional file 3: Movie S1 (right panel)).

### hPARM-1 co-localizes with α-tubulin

Observing the cells incubated at 37°C, we found that hPARM-1-GFP travels in a linear fashion, most likely along the microtubules. When transfected NIH/3T3 cells were stained with the anti-α-tubulin antibody, we showed that some vesicles clearly localized along the microtubule cytoskeleton (Rr: 0.33) (Figure 4l). When treated with nocodazole, cells expressing hPARM-1-GFP showed a drastic inhibition of vesicular movement and a more pronounced hPARM-1-GFP expression at the cell surface (Additional file 4: Movie S2). These results emphasize the important role of tubulin network in hPARM-1 trafficking and demonstrate that its destabilization leads to PARM-1-GFP accumulation at cell periphery.

### PARM-1 colocalizes with caveolin-1

The subcellular localization of the hPARM-1-GFP and caveolin-1 was determined in NIH/3T3 cells. We found that hPARM-1 and caveolin-1 proteins co-localized at the plasma membrane as well as in a few intracellular vesicular pools (Rr: 0.72) (Figure 4m). This result was also confirmed using the ∆CT-GFP mutant which also co-localized with caveolin-1 (Rr: 0.74) (Figure 4n).

### PARM-1 enhances proliferation and serum-independent growth

**Figure 5:**
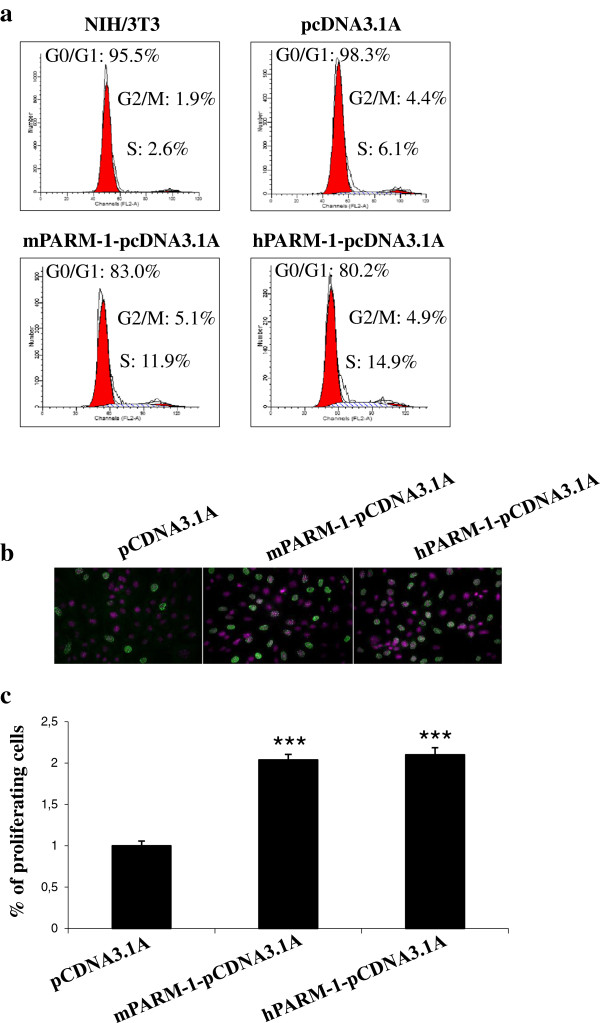
**Effect of mPARM-1 and hPARM-1 on cell cycle of NIH/3T3 cells. (a)** Synchronized NIH/3T3 cells were either untransfected, or transfected with the empty vector (pcDNA3.1A/Myc-His), murine or human Parm-1 constructs. Cells were fixed at 72h post-transfection, stained with propidium iodide and analyzed for cell cycle phase distribution. Percentages of cells in different phases of cell cycle were determined with the ModFit software. **(b)** Proliferation of control pcDNA3.1A/Myc-His, mParm-1-pcDNA3.1A or hParm-1-pcDNA3.1A transfected NIH/3T3 cells was analyzed using BrdU, 48h after transfection. DAPI labeled nuclei are in purple and cells that have incorporated BrdU are in green. Representative fields were photographed. **(c)** The percentage of proliferating cells was determined using the following formula: % of proliferating cells = (number of BrdU incorporating cells/total number of DAPI stained cells) * 100. Values were normalized relative to control cells. For **a**, **b** and **c**, similar results were obtained using either PARM-1 tagged GFP or Myc-His (data not shown). All results represent the average of three independent experiments. Statistical analysis was performed using one-way analysis of variance (*P ≤ 0.05, **P ≤ 0.01, ***P ≤ 0.001).

Transfected NIH/3T3 cells were tested for cell-cycle progression by FACS analysis. We found that the percentage of NIH/3T3 cells transfected with *mParm-1* or *hParm-1* in S phase is enhanced by 2 fold compared to control cells (Figure 5a). Also, BrdU incorporation in NIH/3T3 cells transfected with either mParm-1-pcDNA3.1A or hParm-1-pcDNA3.1A was 50% higher than that of controls (Figure 5b and 5c) suggesting that PARM-1 is a positive cell-cycle regulator.

**Figure 6:**
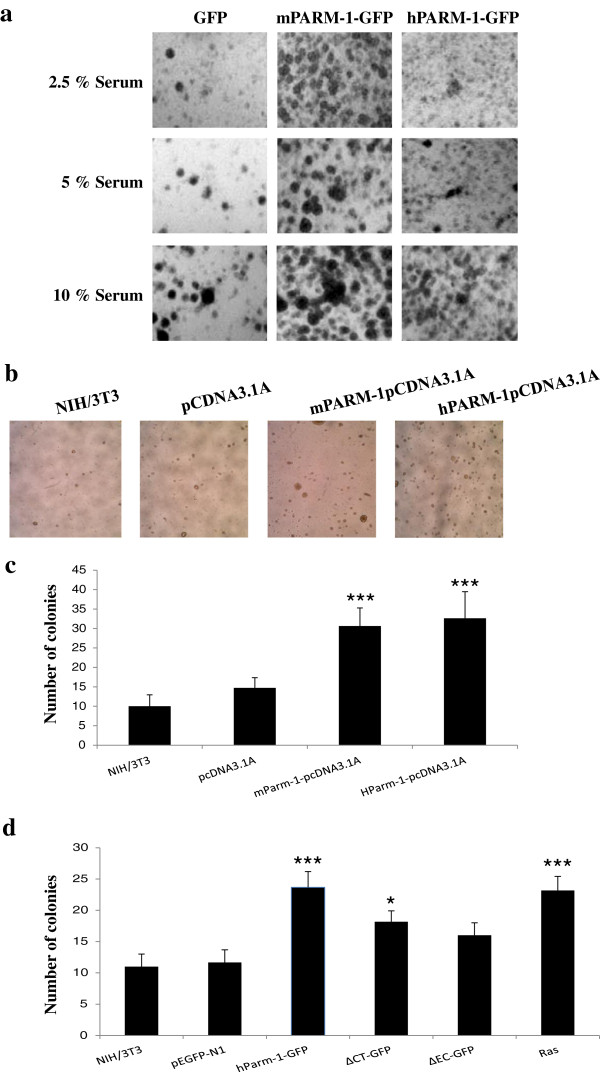
**Effect of mPARM-1 and hPARM-1 on proliferation and on anchorage-independent growth of NIH/3T3 cells. (a)** NIH/3T3 cells were transfected with pEGFP-N1 (GFP), mParm-1-GFP and hParm-1-GFP expression vectors. After 48h post-transfection, 1x10^4^ cells were resuspended in medium containing 2.5%, 5.0% or 10% of bovine serum. Medium was changed every two days. Cells were fixed and stained after 5 days with 0.2% methylene blue and 50% methanol and photographed at 40X magnification. **(b)** NIH/3T3 cells were either untransfected or transfected with pcDNA3.1A/Myc-His empty vector, mParm-1-pcDNA3.1A or hParm-1-pcDNA3.1A expression vectors. Cells (5.10^3^) were plated in soft agar as described in «Colony formation in soft agar». After three weeks, cells were observed with an optical microscope (Ernst Leitz, 6MBH Wetzlar) and representative fields were photographed using a numerical camera (Nikon coolpix 4500; original magnification x40). **(c)** For each image, the number of colonies formed in soft agar was scored using NIH ImageJ software Version 1.42l. **(d)** Cells were untransfected or transiently transfected with pEGFP-N1 empty vector, hParm-1-GFP, ∆CT-GFP and ∆EC-GFP expression vectors. As a positive control, activated Ras (EJ 6.6) expression vector was used. For b and c, similar results were obtained using either PARM-1 tagged GFP or Myc-His (data not shown). The same experiment was done as for full-length constructs. All results represent the average of three independent experiments. For panels **(c)** and **(d)**, the number of colonies in transfected cells was compared to untransfected cells and statistical analysis was performed using one-way analysis of variance (*P ≤ 0.05, **P ≤ 0.01, ***P ≤ 0.001).

Over-expression of either mPARM-1 or hPARM-1-GFP in NIH/3T3 cells grown in the presence of 2.5%, 5% or 10% serum concentrations promoted cell-proliferation compared to control (Figure 6a) indicating that PARM-1 proteins mediate induction of serum-independent cell growth of NIH/3T3.

### PARM-1 protein induces anchorage-independent growth

Classical assay of anchorage-independent growth was performed. We noted that colonies formed in soft agar were much more abundant in both mPARM-1 and hPARM-1-expressing cells compared to controls (Figure 6 be and 6c). Similar result was obtained when GFP-tagged proteins were used (data not shown). These results suggest that both PARM-1 conferred anchorage independence to NIH/3T3 cells.

To identify which portion of hPARM-1 protein could be involved in its oncogenic effect, ∆CT-GFP, ∆EC-GFP and hPARM-1-GFP constructs were used. As a positive control, cells were transfected with the human Ras oncogene (EJ 6.6). Surprisingly, both ∆CT-GFP and ∆EC-GFP mutants increased the number of colonies in soft agar when compared to control cells (Figure 6d). This increase was however lower than that obtained with hPARM-1-GFP in particular for ∆EC-GFP (P < 0.057). These results suggest the importance of the TM-domain and probably a cooperative relationship between the EC- and CT-domains of hPARM-1.

It is important to note that the transient transfection efficiencies in Figures 5 and 6 are ~50%, and therefore the effects observed are actually underestimates of the ability of PARM-1 to change cell growth properties.

### PARM-1 protein over-expression modulates ERK1/2, AKT, and STAT3

**Figure 7:**
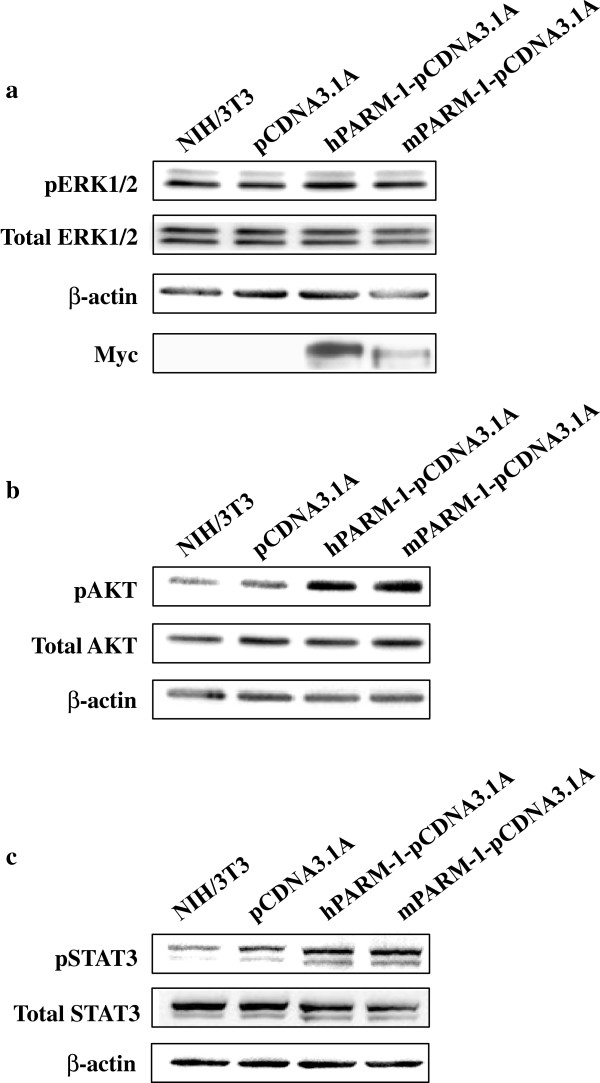
**mPARM-1 and hPARM-1 proteins activate ERK1/2, PI3K/AKT, and STAT3 signaling pathways in NIH/3T3 cells.** NIH/3T3 cells were either untransfected or transfected with empty vector, hParm-1-pcDNA3.1A/Myc-His, mParm-1-pcDNA3.1A, respectively. After 48 h, cell lysates were extracted with specific buffer containing proteinase and phosphatase inhibitors and 30 μg of proteins were resolved by 12% SDS-PAGE. Following protein transfer, PVDF membranes were probed with **(a)** anti-phospho-ERK1/2 (Thr202/Tyr204), **(b)** anti-phospho-AKT or **(c)** anti-phospho-STAT3 (All from cell Signaling; 1:1000) antibodies. Loading of equal amount of protein was verified using anti-anti-p44/42 MAPkinase (ERK), anti-AKT, anti-STAT3 (All from cell Signaling; 1:1000) and anti-β-actin antibodies. Expression of murine and human PARM-1 proteins was verified using an anti-Myc antibody (exemplified with only one membrane). Experiments were performed in triplicate.

We showed that both PARM-1 proteins promote NIH/3T3 cells proliferation but the implication of a specific pathway by this protein remains to be determined. Activations of ERK1/2 [20], AKT [21] and STAT3 [22] dependent signaling pathway are often linked to cell proliferation. The analysis of the phosphorylation levels of ERK1/2, AKT and STAT3 in cell lysates from NIH/3T3 fibroblasts overexpressing mPARM-1 or hPARM-1 showed an up-regulation of their phosphorylation state (especially for AKT and STAT3) (Figure 7a-c) indicating that PARM-1 affect and activate the ERK1/2, AKT, and STAT3 dependent signaling pathways.

## Discussion

The raw microarrays results obtained in our previous microarrays analysis were reanalyzed focusing on genes that were specifically deregulated in T-CD8^+^ leukemias when compared to T-cells control. From this analysis 50 probsets were selected (Fold-change of 4). Some of these genes were already known to be involved in T-CD8^+^ leukemias: *Il2ra* (expressed in primary leukemia cells from a patient with T-CD8^+^ prolymphocytic leukemia) [5]. Our microarray analysis also showed that some other genes were known to be associated with other T-leukemia sub-types or cancer as *Irf4* (an oncogene locus which is frequently translocated in peripheral T-cell lymphomas) [7], *Depdc6* (when over-expressed, it increases survival of hepatocellular carcinoma cells) [9] and *Als2cl* (a tumor-suppressor gene that contributes to the tumorigenesis of head and neck squamous cell carcinoma) [10]. These results validate our new microarray analysis. More interestingly, we also found other genes that had never been associated with leukemias nor with other types of cancer, or had no assigned function such as the *Exoc3l4*[23], *Hectd2*[24,25] and AU014947. The complete list of these genes, which are good candidates for specific markers, oncogenes or tumour suppressors for T-CD8^+^ leukemias, is presented in Table 1.

From this list, we focused on the *9130213B05Rik* that corresponds to the conserved *mParm-1* gene (Additional file 1: Figure S1) and we validated the specificity of its over-expression in Graffi MuLV induced T-CD8^+^ tumors (Figure 1).

Our interest for this gene was drained by the fact that *Parm-1* was poorly characterized and had never been clearly associated with cancer. Indeed, the rat *Parm-1* is over-expressed in prostate epithelial cells after androgen deprivation following castration [26]. However, its human counterpart expression is increased by androgen in the LNCaP prostate cancer cell line and decreased in the CWR22 xenograft upon castration [17]. Moreover, ectopic expression of *hParm-1* in human prostate cancer cell line enhances their proliferation [17]. However, the rat *Parm-1* had no effect on rat cancer cell line [27]. In contrast, even if *in vivo* models demonstrated that over-expression of *Parm-1* is not implicated in apoptosis [26], *in vitro* models suggested that *Parm-1* is indirectly involved in the survival program [27]. Also, it was demonstrated that *Parm-1* silencing in rat cardiac myocytes enhanced apoptotic response to endoplasmic reticulum stress [28]. Due to these conflicting data, we further characterized the function and determined the oncogenic potential of PARM-1.

The human mucin family can be sub-classified into secreted and membrane-associated mucin forms [14,29,30]. The extracellular domain of most transmembrane mucins is released from the cell surface [14]. Since PARM-1 shares similar structure with the membrane-associated mucins (Figure 2a and 2b), we determined whether the EC-domain of this highly conserved protein is also released. We showed that hPARM-1 is weakly intact secreted protein (Figure 3 and Additional file 2: Figure S2). This result, although unexpected for proteins of the mucin family, correlates with data reported for many other type I transmembrane proteins such as APP [31], N-CAM [32], insulin receptor [33], recombinant EGF precursor [34], and c-Kit receptor proto-oncogene [35].

Our results for PARM-1 subcellular localization agree with previous report [17], for hPARM-1 and extend our observations to the mPARM-1. Indeed, we show that both proteins co-localized within the Golgi and at early and late endosomes but weakly localized at the plasma membrane (Figure 4). The same localization was observed in NIH/3T3 cells transfected with ∆EC-GFP and ∆SP-GFP mutants (Figure 4g and 4h). However, EC-GFP and ∆TM-GFP mutants showed a GFP-like localization (Figure 4i and 4j) and ∆CT-GFP mutant predominantly showed plasma membrane localization (Figure 4k). These results suggest that TM probably determines the Golgi-endocytic pathway localization. Such observation had already been reported for other proteins as the type I transmembrane BACE1 protein. BACE1 is mainly located in the distal Golgi membrane but not considerably present at the plasma membrane of neuroblastoma cells. It was demonstrated that the TM-domain determines its Trans-Golgi Network (TGN) localization [36]. Our results also suggest that CT-domain inhibited plasma membrane localization (Figure 4k). This is reinforced by the fact that mutations in the CT (_287_YGRL_290_ to _287_AGRA_290_) induced PARM-1 plasma membrane localization [17]. This YGRL motif acts as a tyrosine-based plasma membrane internalization signal [37] also present in Syntaxin-6 (STX6) protein which is localized to the TGN. Importantly, it was demonstrated that deletion of this motif prevents STX6 internalization and induces its plasma membrane accumulation [38]. Our data suggest that YGRL motif induces hPARM-1 internalization. Indeed, we showed that the internalization process of hPARM-1 was temperature-dependent, very dynamic at 37°C and dramatically inhibited at 4°C (Additional file 3: Movie S1). These results suggest a very quick internalization for hPARM-1 and may explain that the protein remains barely detectable at the plasma membrane.

It has been established that endosomes and endocytic proteins can traffic via microtubules [39,40]. Our data indicated the important role of microtubules in PARM-1 trafficking. In fact, PARM-1 co-localized with the microtubule cytoskeleton (Figure 4l) and depolymerisation of its network with nocodazole induced a dramatic inhibition of PARM-1 trafficking accompanied by an accumulation of an important portion of PARM-1 at the cell periphery (Additional file 4: Movie S2). We also found that hPARM-1 co-localized with caveolin-1 (Figure 4m). This preliminary result suggests that PARM-1 internalization may be mediated via the caveolae. Further investigations will be needed to confirm the involvement of caveolin-1 in this process.

It is known that mucins are implicated in cancer development [29] but there were no convincing data yet on the role of *Parm-1* in cellular transformation. We showed that PARM-1 enhanced the proliferative capacities (Figure 5) and confer the serum-independent growth to NIH/3T3 cells (Figure 6a) suggesting that it could induce an autocrine loop in cells thus stimulating their proliferation in absence of growth factors. Using the classical NIH/3T3 colony formation in soft agar test, we demonstrated that ectopic expression of PARM-1 conferred anchorage-independent growth to the cells and we found that both deletion mutants (∆CT-GFP and ∆EC-GFP) seem to retain part of their ability to confer this capacity to the cells (Figure 6b-d). These results let us speculate that the TM-domain should play an important role in the protein function especially in its targeting toward the appropriate cell compartment. It also suggests a complementary or collaborative role for EC- and CT-domains, respectively, with TM to induce anchorage independence. Similar results were reported for the MUC1 protein where EC- and CT-domains contribute separately to the cancer cell line invasiveness and metastasis [41].

We also analyzed the downstream signaling events leading to proliferation and provided first evidence on the role of PARM-1 in ERK1/2 and especially in AKT and STAT3 dependent signaling pathways (Figure 7). These pathways are a part of a more complex process leading to cell proliferation enhancement. In fact, the AKT is implicated in cell survival, growth and proliferation [21]. ERK1/2 is also implicated in the cell proliferation. Interestingly, these two pathways are constitutively activated in several human cancers [20]. Moreover, it is known that the STAT3 Ser-727 is phosphorylated by ERK1/2 [42,43] and that STAT3 is also implicated in the proliferation tumor-derived cell lines [44]. In summary, activation of ERK1/2, AKT, and STAT3 shed further light on the mechanism by which PARM-1 may contribute to transformation.

## Conclusions

Overall, our results strongly support an oncogenic role for *Parm-1*, member of the mucin family, especially in T-CD8^+^ leukemia and enable us to propose the following model: newly synthesized protein accumulates to the Golgi where post-transcriptional modifications occur (glycosylation and probably dimerization). A major fraction of PARM-1 protein will be retained in this compartment via its TM-domain, which seems to play a determinant role in the oncogenic potentiality of the protein. Certain amount of the protein will be packaged in vesicles for transport to the plasma membrane where a minor fraction of the entire PARM-1 will be secreted and could serve as a ligand (unknown receptor), which in turn leads to the activation of the downstream signaling pathway. In parallel, the YGRL motif will induce the rapid internalization and recycling of the intracellular protein, a prerequisite for its activity indicating that non-secreted PARM-1 could act as a new receptor or transporter. These data suggest a complex role for PARM-1. Further studies are required to better understand PARM-1 functions and could provide new tools to develop new therapeutic approaches in the treatment of human cancer.

## Methods

### Mice sample collection and flow cytometry

To generate leukemias, newborn NFS, FVB/n or Balb/c mice were injected intraperitoneally with GV-1.4 (1. 10^6^ PFU) or GV-1.2 (3. 10^6^ PFU) viral particles [1]. Moribund mice were sacrificed. Lymph nodes, thymus, bone marrows and spleens were harvested for flow cytometry analysis [1,3] and RNA extraction [3]. All the experimental procedures were approved by the Animal Care Committee of Université du Québec à Montréal.

### Microarrays and gene expression analysis

Using the microarrays data set normalized from our anterior study [3], the RMA values of the 45000 probsets were used to identify differentially expressed genes in T-CD8^+^ leukemias. Genes were selected according the following criteria : the expression signal intensity did not vary in B leukemias versus control B-cells and the expression signal intensity was either significantly higher (up-regulated), or lower (down-regulated) in T-CD8^+^ leukemias versus control cells (fold-change of 4). The microarray dataset was deposited at Gene Expression Omnibus under the accession number GSE12581 [3].

### Semi-quantitative RT-PCR

Total RNA (100 ng) was reverse transcribed using the Omniscript enzyme (QIAGEN) and the oligo(dT) primer. The semi-quantitative PCR reactions were performed with the Taq polymerase kit (Feldan) using an RT reaction corresponding to 10 ng of RNA samples and to 2 ng for actin, (94°C for 3 min, 94°C for 45 s, 56°C for 45 s, 72°C for 30 s with a final extension at 72°C for 10 min). Annealing temperature and number of cycles were optimized for each gene.

### Plasmid constructions

The cDNA of the complete coding region of *mParm-1* and hParm*-*1 were generated by standard PCR amplification method using primers containing specific restriction sites. The PCR products were then inserted in-frame within the pEGFP-N1 (Clontech Laboratories) or pcDNA3.1/Myc-His(+)A (Invitrogen) vectors. Deletions were generated using specific primers that amplify the specific region of interest and the PCR products inserted in-frame in pEGFP-N1.

### Cell culture

NIH/3T3 and Jurkat T-cells were obtained from ATCC (Rockville). NIH/3T3 cells were grown in DMEM medium supplemented with 10% CS and Jurkat cells were cultured in RPMI supplemented with 10% FCS (Invitrogen). 50 U penicillin and of streptomycin (Gibco, Invitrogen) were added.

### Confocal microscopy

For transient transfection, Jurkat cells (10^7^) were transfected with 15 μg plasmids by electroporation with the Gene Pulser System (Bio-Rad). NIH/3T3 cells were transfected using the polyfect reagent (Qiagen). Both pEGFP-N1 (control) and GFP-tagged *mParm-1* or *hParm-1* genes were used.

Localization of mPARM-1 and hPARM-1 was performed by confocal microscopy 48 h after transfection. For cell surface membrane co-localization (CellMask™ Plasma Membrane Stains (Invitrogen)), Jurkat cells were pelleted 48 h after transfection, washed in PBS and overlaid for 30 min at 37°C on polylysine coated glass slides [3]. For co-localization experiments, NIH/3T3 cells were plated on glass coverslips, grown at 50% confluency, and transfected as described above. After 48 h of transfection, cells were fixed with 4% paraformaldehyde, followed by PBS washes and permeabilization with 0.1% Triton X-100 in PBS. Cells were blocked in PBS with 10% goat serum, 10% BSA and 0.1% triton, and incubated with primary antibodies. Coverslips were incubated with Alexa-Fluor-568-conjugated secondary antibody (1/1000, Invitrogen), washed with PBS, mounted onto slides using Prolong Gold antifade reagent (Invitrogen) and observed by confocal microscopy.

For live cell imaging, cells were transfected and sub-cultured into dishes containing glass coverslip. After 48 h, glass coverslips were transferred to coverslip-cell chamber and maintained at 37°C or at room temperature if cells were previously incubated at 4°C before imaging.

### Western blot analysis

NIH/3T3 cells were homogenized in lysis buffer (50 mM Hepes, pH 7.5, 150 mM NaCl, 10 mM sodium pyrophosphate, 100 mM sodium fluoride, 1.5 mM MgCl2, 1 mM EGTA, 200 μM sodium orthovanadate, 1 mM phenylmethylsulfonyl fluoride, 10% glycerol, and 1% Triton X-100) supplemented with a complete protease inhibitor cocktail (Roche) and phosphatase inhibitors (Sigma). Cells were incubated for 30 min at 4°C, and centrifuged at 15,000 X g for 10 min at 4°C.

For secretion experiment, NIH/3T3 supernatant was collected, centrifuged at 500 X g for 5 min and concentrated twenty times with a 10 kDa cut-off Amicon Ultra (Millipore). Secreted and cell lysate proteins were separated on SDS-PAGE and transferred to PVDF membrane. Membranes were blocked in buffer (PBS, 0.1% Tween 20 [PBS-T] with 5% nonfat dry milk) and incubated overnight at 4°C with primary antibodies. Membranes were incubated with horseradish peroxidase–conjugated secondary antibodies diluted in blocking buffer and signal was revealed by Immobilon Western HRP reagent (Millipore).

### Colony formation in soft agar

To determine the anchorage-independent growth, colony formation was tested in soft agar as previously described [3,45]. Briefly, NIH/3T3 cells were transiently transfected with the empty vector (pcDNA3.1A/Myc-His or pEGFP-N1), Ras EJ 6.6, mParm-1-pcDNA3.1A or mParm-1-GFP and hParm-1-pcDNA3.1A or hParm-1-GFP expression vectors. After 48 h, cells were mixed with melted 0.3% agarose in DMEM medium and seeded on top of a 0.6% agarose base layer containing the same medium. Cells were fed twice a week for 4 weeks and observed with an optical microscope.

### Cell cycle analysis

Flow cytometry was performed using a FACScan flow cytometer (Becton Dickinson). Briefly, 1x10^6^ cells were pelleted, resuspended in 0.2 ml of PBS, added to ice-cold 70% ethanol and incubated overnight at 4°C. Cells were pelleted, resuspended in propidium iodide (40 μg/ml)-RNase (100 μg/ml) solution for 30 min at 37°C and analyzed by flow cytometry for their DNA content.

### Bromodeoxyuridine (BrdU) incorporation

BrdU incorporation was monitored using a 5-Bromo-2’-deoxy-uridine labeling and Detection kit I (Roche). Briefly, 48h transfected cells were incubated in the presence of BrdU, fixed with ethanol, washed with PBS and incubated with mouse monoclonal anti-BrdU antibody (clone BMC 6H8). Cells were incubated with an anti-mouse immunoglobulin-fluorescein antibody solution. Cells were incubated in a solution of DAPI (15 000), mounted onto slides using Prolong Gold antifade reagent (Invitrogen) and observed by fluorescent microscopy.

### Cell growth in low serum conditions

NIH/3T3 cells were transiently transfected as mentioned above and 48 h later, cells were seeded at a low density in DMEM containing 2.5%, 5% or 10% CS for 5 days. Cells were fixed, stained and photographed.

## Competing interests

The authors declare that they have no competing interests.

## Authors’ contributions

CC carried out the design and coordination of the study, performed all experiments and drafted the manuscript. LLCJ helped to Parm-1 cloning. EE and ER contributed to the interpretation of the data and helped to write the manuscript. All authors read and approved the final manuscript.

## Supplementary Material

Additional file 1: Figure S1 Alignment of deduced amino acid sequences for eight species of PARM-1 protein. Alignment of Homo sapiens (NP_056208_2), Mus musculus (NP_663537_1), Pan troglodytes (XP_001155067_1), Pongo abelii (NP_001127394_1), Sus scrofa (NP_001230645_1), Bos taurus (NP_001069239_1), Rattus norvegicus (NP_775137_1), and Gallus gallus (XP_429964_2) proteins. The most conserved amino acids are represented in red and the unconserved one in blue. A color code is presented in the top of the figure. Click here for file

Additional file 2: Figure S2 PARM-1 protein profile and secretion by NIH/3T3 cells. Immunoblotting of lysates (30 μg) from NIH/3T3 cells transiently transfected with expression vector containing full-length hParm-1 or mutants using (a) an anti-hPARM-1 antibody (Sigma; 1:1000) or (b) an anti-GFP antibody (Santa Cruz Biotechnology; 1:1000). Culture supernatants from these cells were collected, centrifuged, concentrated and subjected to SDS-PAGE (12%) and hPARM-1 protein was detected by western blot as above. Two exposures are presented. Click here for file

Additional file 3: Movie S1 Temperature effect on hPARM-1-GFP vesicles trafficking. NIH/3T3 cells were transfected with hParm-1-GFP construct. After 48h, living cells were either maintained at 37°C (left panel) or incubated for 1h at 4°C (right panel). Cells were then analyzed by confocal microscopy and images were collected at a rate of 2 seconds over a period of 10 min. Click here for file

Additional file 4: Movie S2 Effect of microtubule depolymerization on the trafficking and localization of hPARM-1 protein. NIH/3T3 cells expressing hPARM-1-GFP protein were incubated for 30 min with 1 μg/ml nocodazole (an agent interfering with microtubules polymerization) to disturb the microtubulin cytoskeleton. Cells were then analyzed by confocal microscopy. Images were taken at a rate of 2 seconds over a period of 10 min. Click her for file
