# Supplementary material for: Characterization and identification of PARM-1 as a new potential oncogene
Source: Mol Cancer. 2013 Jul 31;12:84. doi: 10.1186/1476-4598-12-84 (PMC3750824; doi:10.1186/1476-4598-12-84)
Supplement: Additional file 1: Figure S1 — Alignment of deduced amino acid sequences for eight species of PARM-1 protein. Alignment of Homo sapiens (NP_056208_2), Mus musculus (NP_663537_1), Pan troglodytes (XP_001155067_1), Pongo abelii (NP_001127394_1), Sus scrofa (NP_001230645_1), Bos taurus (NP_001069239_1), Rattus norvegicus (NP_775137_1), and Gallus gallus (XP_429964_2) proteins. The most conserved amino acids are represented in red and the unconserved one in blue. A color code is presented in the top of the figure. [file 1476-4598-12-84-S1.pdf]

Unconserved 0 1 2 3 4 5 6 7 8 9 10 **Conserved**

```
                   .........10 .........20 .........30 .........40 .........50
Homo sapiens       -----MVYKT LFALCILTAG WRVQSLPTSA PLSVSLPTNI VPPTTIWTSS
Pan troglodytes    -----MVYKT LFALCILTAG WRVQSLPTSA PLSVSLPTNI MPPTTIWTSS
Mus musculus       -----MVCKV LIALCIFTAG LRVQGSPT-V PLPVSLMTKS SAPVATWTTS
Pongo abelii       -----MVYKT LFALCILTAG WRVQSLPTSA PLSVSLPTNI MPPTTIWTSS
Sus scrofa         -----MVCKT LFALFIVTAG LRVQRVSAST VLPVFLPTKI TPATAVWTSP
Bos taurus         -----MVCKT LFALCIFTAG LRLQSVSASI PSTDSLLAET TTPTAIWTSS
Rattus norvegicus  -----MVCKA LITLCIFAAG LMVQGSPTPT LLPVSLTTKS TAPMATWTTS
Gallus gallus      MHPDLAVRPA ALVPCRYYQK LSDSSWPAAL PALQDSYEFA PDFQADHGGA
Consistency        000008*486 8677775688 5668647755 6646683745 4466757867

                   .........60 .........70 .........80 .........90 .........100
Homo sapiens       PQNTDADTAS PSNGTHNNSV LPVTASAPTS LLPKNISI-- -ESREEEITS
Pan troglodytes    PQNTDADTAS PSNGTHNNSV LPVTASAPTS LLLKNISV-- -ESREEEITS
Mus musculus       APHTARATTP VASATHNASV LRTTAASLTS ----QLPT-- -DHREEAVTS
Pongo abelii       PQNTDADTAS PSNGTHNNSV LPVTASAPTS LLPKNISV-- -ESREEEITS
Sus scrofa         PQSPPASPTS GTPSTSVLPI IASAPTSPPT --KNVSVE-- -AGAEEEPTS
Bos taurus         PQ---SPPAS PTSGPSNSSV LLDPTPVPTS LPTKNIST-- -EPREEQSTS
Rattus norvegicus  AQHTAMATTP VASATHNASV LRTTAASLTS ----QLPT-- -HPREEAVTS
Gallus gallus      AGLKDTQTPG LGDDPVAPSL APFPVVGTSA GPGSGGTPVP TAAPHQSTPL
Consistency        6634354766 4655756488 7446655578 2113455400 0546896588

                   .........110.........120.........130.........140.........150
Homo sapiens       PGSNWEGTNT DPSPSGF--- SSTSGGVHLT TTLEEHSSGT PEAGVAATLS
Pan troglodytes    PGSNWEGTNT DPSPPGF--- SSTSGGVHLT TTLEEHSSGT PEVGMAATLS
Mus musculus       PPLKRDVNST DSSPAGF--- PSTSSDGHLA PTPEEHSLGS PEATVPATGS
Pongo abelii       PGSNWEGTNT DPSPPGF--- SSTSGGVHLT TTLEEHSLGT PEAGVAATLS
Sus scrofa         PASQSAGTET VPSSTSG--- GLH-----LT PTPEEHSPVT PEASVPATGS
Bos taurus         PASNWEGTDP SPTSGGV--- H-------LT PTPEEHSSDT PEAGVPTTGS
Rattus norvegicus  PPLKREVNST DSSPTGF--- SSNSSGIHLA PTPEEHSLGS PETSVPATGS
Gallus gallus      TAVGPTSNGV SPEVGDRNGS TTAMLSRAST LTNTVTAADS PSVAPSSVPP
Consistency        8555365657 5775474000 4544232387 5*48879457 *865757838

                   .........160.........170.........180.........190.........200
Homo sapiens       QSAAEPPTLI SPQAPASSPS SLST--SPPE VFSASVTTNH SSTVTSTQPT
Pan troglodytes    QSTAEPPTLI SPQAPASSPS SLST--SPPE VFSASVTTNH SSTVTSTQPT
Mus musculus       QSPML----L SSQAPTSATT SPAT--SLSE SLSASVTSSH NSTVANIQPT
Pongo abelii       QSAAEPPTLI SPQAPASSPS SLST--SPPE VFSVSVTTNH SSTVTSTQPT
Sus scrofa         QPQAESPALT SPQAPTSSPS PPST--SPLE VPSASISTSN SSTETSTKPT
Bos taurus         QPPAESPTLT SPQGPASSPL PPST--SPPE VPSASISTSH SSAETSTEPT
Rattus norvegicus  QSPTL----L FSQGPTSAST SPAT--SPSE PLSASVTSNH SSTVNNIQPT
Gallus gallus      TLETVLSPWT AHSSVARGTT DLGTNPSPVD TPTSFSSSSP HNSTLHSSPG
Consistency        8546524336 7586878756 647*00*749 5387877776 7875666 7*8

                   .........210.........220.........230.........240.........250
Homo sapiens       GAPTAPE--S PTEESSSDHT PTSHATAEPV -PQEKTPPTT VSGKVMCELI
Pan troglodytes    GAPTAPE--S PTEESSSDHT PTSHATAEPV -PQEKTPPTT VSGKVMCELI
Mus musculus       EAPMAPASPT EEHSSSHTPT SHVTAEPVPK -EK--SPQDT EPGKVIC---
Pongo abelii       GAPTAPE--S PTEESSSDHT PTSHATAEPV -PQEKTPPTT VSGKVMCELI
Sus scrofa         GAPTTPKSPE EEHSSGQTPT SHATATPMPT -ET--TSQAA VPPKGTPMLI
Bos taurus         GAPTTPESHT EEHSSTLTPT SHASSESVPT -EA--TPQAT VPPKVTCILI
Rattus norvegicus  GAPMAPA--- SPTEEHSSSH TPTSHVTEPV -PKEKSPQDT EPGKVIC---
Gallus gallus      TALLSPAIPT QPPGLTKDVP SPGTLAVAPS LAMEPTSPSV TAASPTKGMA
Consistency        6*767*5114 5446654536 54555544*5 0543287657 5558655144

                   .........260.........270.........280.........290.........300
Homo sapiens       DMETTTTFPR VIMQEVEHAL SSGSIAAITV TVIAVVLLVF GVAAYLKIRH
Pan troglodytes    DMETTTTFPR VIMQEVEHAL SSGSIAAITV TVIAVVLLVF GVAAYLKIRH
Mus musculus       --ESETTTPF LIMQEVENAL SSGSIAAITV TVIAVVLLVF GGAAYLKIRH
Pongo abelii       DMETTTTFPR VIMQEVEHAL SSGSIAAITV TVIAVVLLVF GVAAYLKIRH
Sus scrofa         DTETTAASPR VIMQEVEHAL SSGSIAAITV TVIAVVLLVF GVAAYLKIRH
Bos taurus         DMETTTTSPG VIMQEVEHAL SSGSIAAITV TVIAVVLLVF GVAAYLKIRH
Rattus norvegicus  --ESETTTPF LIMQEVENAL SSGSIAAITV TVIAVVLLVF GAAAYLKIRH
Gallus gallus      EEGKSTPSTG VTIEEVPHAL SAGSIVAITV TVIVVVVLVF GAAAYLKIRH
Consistency        4386687483 8889**87** *9***8**** ***8**9*** *6********

                   .........310.........320........
Homo sapiens       SSYGRLLDDH DY--GSWGNY NNPLYDDS
Pan troglodytes    SSYGRLLDDH DY--GSWGNY NNPLYDDS
Mus musculus       SSYGRLLDDH DY--GSWGNY NNPLYDDS
Pongo abelii       SSYGRLLDDH DY--GSWGNY NNPLYDDS
Sus scrofa         SSYGRLLDDH DY--GSWGNY NNPLYDDS
Bos taurus         SSYGRLLDDH DY--GSWGNY NNPLYDDS
Rattus norvegicus  SSYGRLLDDH DYGSGSWGNY NNPLYDDS
Gallus gallus      SSYGRLLDDH DY--GSWGNY NNPLYDDS
Consistency        ********** **00****** ********
```
