# Supplementary figures and images for: Characterization and identification of PARM-1 as a new potential oncogene
Source: Mol Cancer. 2013 Jul 31;12:84. doi: 10.1186/1476-4598-12-84 (PMC3750824; doi:10.1186/1476-4598-12-84)

**a**

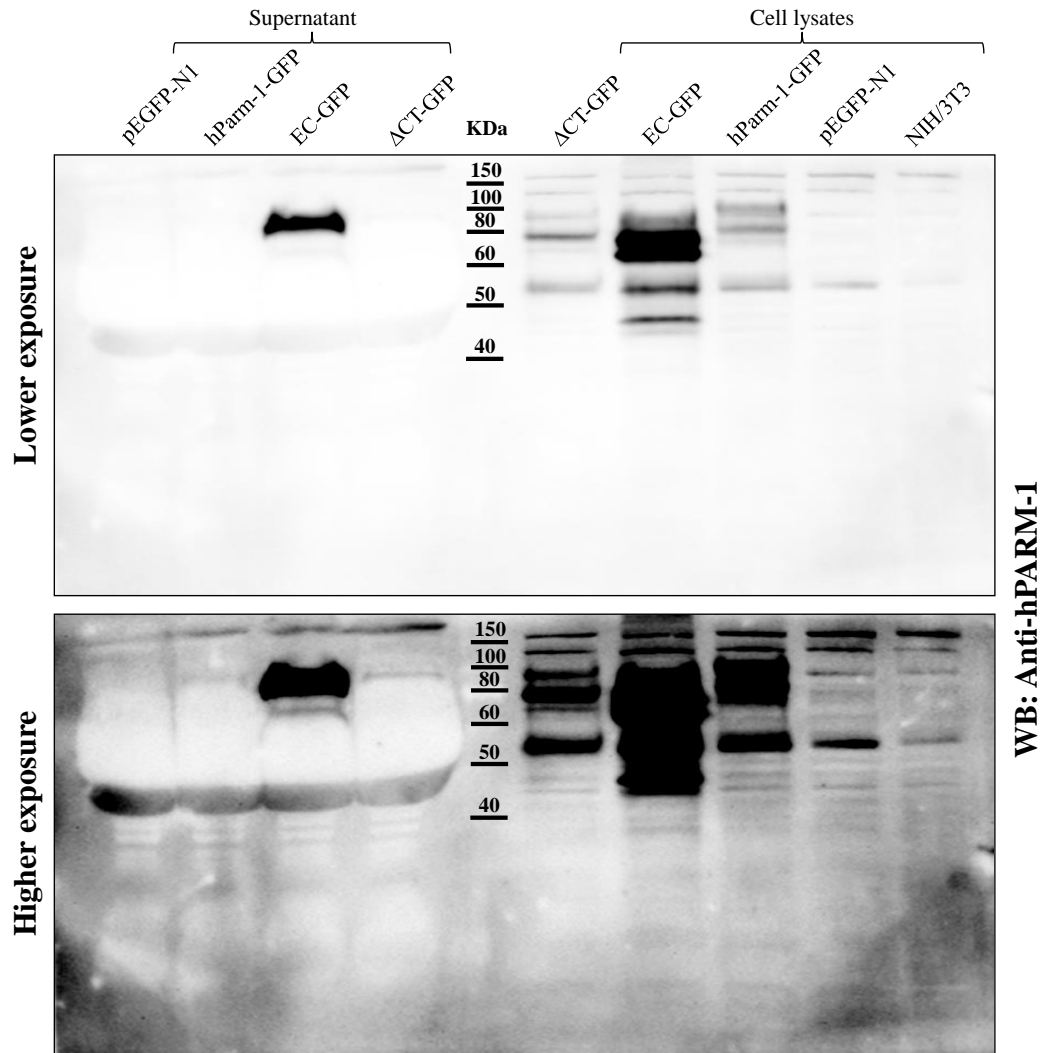

**b**

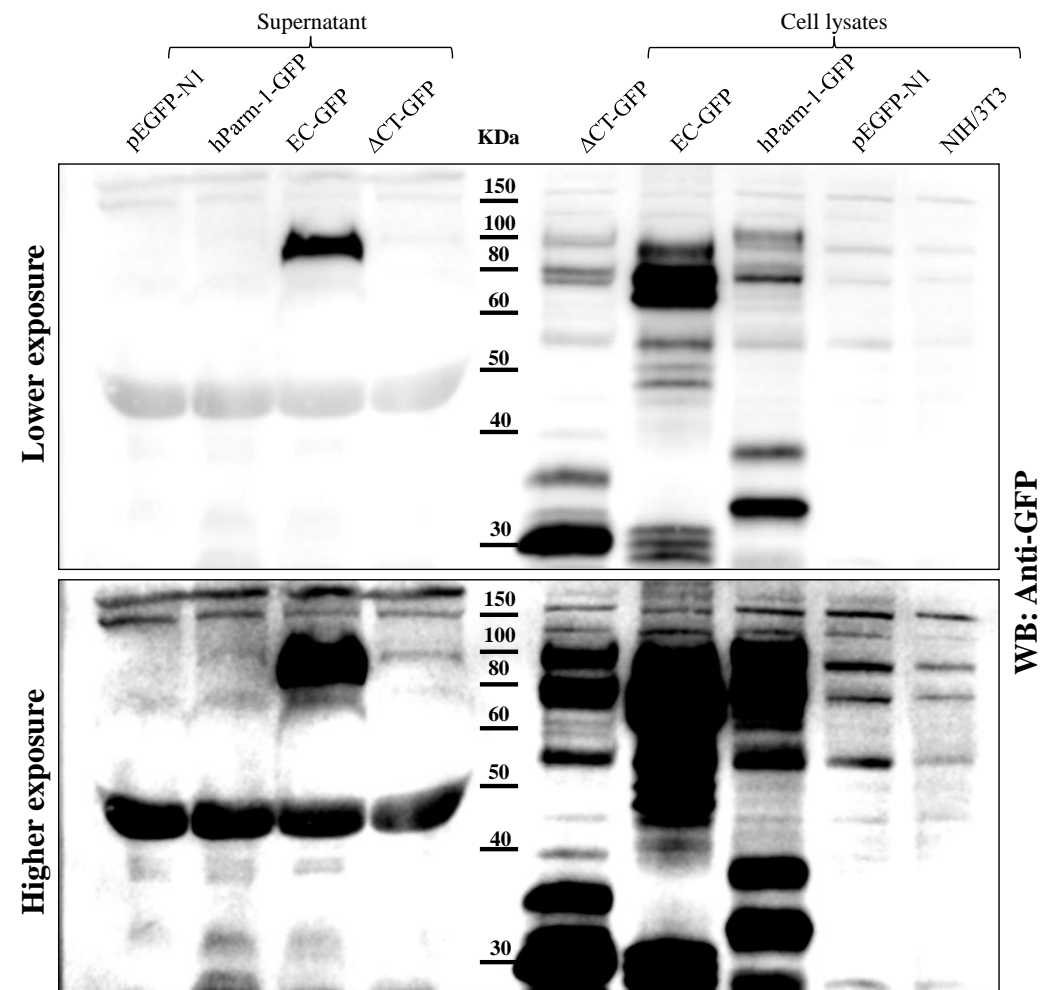

Supplement: Additional file 2: Figure S2 — PARM-1 protein profile and secretion by NIH/3T3 cells. Immunoblotting of lysates (30 μg) from NIH/3T3 cells transiently transfected with expression vector containing full-length hParm-1 or mutants using (a) an anti-hPARM-1 antibody (Sigma; 1:1000) or (b) an anti-GFP antibody (Santa Cruz Biotechnology; 1:1000). Culture supernatants from these cells were collected, centrifuged, concentrated and subjected to SDS-PAGE (12%) and hPARM-1 protein was detected by western blot as above. Two exposures are presented. [file 1476-4598-12-84-S2.pdf]
